# Glioma cell proliferation is enhanced in the presence of tumor-derived cilia vesicles

**DOI:** 10.1186/s13630-018-0060-5

**Published:** 2018-11-06

**Authors:** Lan B. Hoang-Minh, Marina Dutra-Clarke, Joshua J. Breunig, Matthew R. Sarkisian

**Affiliations:** 1 Department of Neuroscience, University of Florida College of Medicine, McKnight Brain Institute, Gainesville, FL 32610 USA; 2 Preston A. Wells, Jr. Center for Brain Tumor Therapy, University of Florida College of Medicine, McKnight Brain Institute, Gainesville, FL 32610 USA; 3 Board of Governors Regenerative Medicine Institute, Cedars-Sinai Medical Center, Los Angeles, CA 90048 USA; 4 Department of Biomedical Sciences, Cedars-Sinai Medical Center, Los Angeles, CA 90048 USA; 5 Samuel Oschin Comprehensive Cancer Institute, Cedars-Sinai Medical Center, Los Angeles, CA 90048 USA

**Keywords:** Primary cilia, Arl13b, Vesicle, Budding, F-actin, Cancer, Proliferation, Smoothened, Glioblastoma

## Abstract

**Background:**

The mechanisms by which primary cilia affect glioma pathogenesis are unclear. Depending on the glioma cell line, primary cilia can promote or inhibit tumor development. Here, we used piggyBac-mediated transgenesis to generate patient-derived glioblastoma (GBM) cell lines that stably express Arl13b:GFP in their cilia. This allowed us to visualize and analyze the behavior of cilia and ciliated cells during live GBM cell proliferation.

**Results:**

Time-lapse imaging of Arl13b:GFP^+^ cilia revealed their dynamic behaviors, including distal tip excision into the extracellular milieu. Recent studies of non-cancerous cells indicate that this process occurs during the G0 phase, prior to cilia resorption and cell cycle re-entry, and requires ciliary recruitment of F-actin and actin regulators. Similarly, we observed ciliary buds associated with Ki67^−^ cells as well as scattered F-actin^+^ cilia, suggesting that quiescent GBM cells may also utilize an actin network-based mechanism for ciliary tip excision. Notably, we found that the proliferation of ciliated GBM cells was promoted by exposing them to conditioned media obtained from ciliated cell cultures when compared to conditioned media collected from cilia-defective cell cultures (depleted in either KIF3A or IFT88 using CRISPR/Cas9). These results suggest that GBM cilia may release mitogenic vesicles carrying factors that promote tumor cell proliferation. Although Arl13b is implicated in tumor growth, our data suggest that Arl13b released from GBM cilia does not mediate tumor cell proliferation.

**Conclusion:**

Collectively, our results indicate that ciliary vesicles may represent a novel mode of intercellular communication within tumors that contributes to GBM pathogenesis. The mitogenic capacity of GBM ciliary vesicles and the molecular mediators of this phenomenon requires further investigation.

**Electronic supplementary material:**

The online version of this article (10.1186/s13630-018-0060-5) contains supplementary material, which is available to authorized users.

## Introduction

Gliomas are the most common primary malignancies of the central nervous system (CNS). Among them, glioblastoma (GBM) is the most prevalent form in adults, conferring the poorest prognosis with a median survival of 15 months following diagnosis. GBM resistance to therapy stems from its heterogeneity and overwhelmingly immunosuppressive tumor microenvironment (for review see [1–3]).

The role of primary cilia in cancer has been generating increasing interest over the past 10–15 years [4, 5]. Primary cilia are microtubule-based organelles which can mediate signaling that influences the proliferation of normal and tumor cells [e.g., through the mammalian Sonic Hedgehog (SHH), platelet-derived growth factor (PDGF), and Notch pathways], including various cancers of the CNS (medulloblastoma, ependymoma, choroid plexus tumors) [6–8]. However, the role primary cilia play in GBM remains poorly understood.

A few studies have examined the presence and structure of primary cilia in glioma. Analyses of some patient biopsies [9] and cultured ‘U’ and ‘T’ glioma cell lines [10] have revealed that primary cilia are largely absent or display ultrastructural defects on those tumor cells. On the other hand, a significant number of primary cilia displaying the ultrastructural properties of normal cilia have been detected in other patient GBM biopsies and more recent patient-derived cell lines [11]. Additionally, in patient-derived lines cell lines that were about 15–25% ciliated, we found that the majority (~ 60–90%) of isolated single clones generated ciliated progeny [12]. The frequency of observed ciliated cells was low (~ 5–20%), which might have been due to the temporary disassembly of cilia required for cell division and cell cycle progression [13–15].

The role primary cilia play during GBM pathogenesis may be dual, as there is evidence that the presence or loss of cilia, or associated signaling pathways, can promote tumor growth. Mutations in the SHH signaling pathway, which primary cilia are known to transduce [16, 17], can promote tumor growth in a fraction of GBMs [18–21]. In addition, we observed that the SHH-induced proliferation of some GBM cells required functionally intact primary cilia [12]. However, suppressing cilia formation in other GBM cell lines, by disrupting critical ciliogenesis genes such as KIF3A or IFT88, had variable effects on tumor growth in vitro and in vivo. Disrupting KIF3A and IFT88 in an SHH-responsive GBM cell line slowed tumor progression, whereas disrupting KIF3A in other SHH non-responsive cell lines either accelerated or had no effect on tumor growth [12]. Moreover, CRISPR/Cas9 ablation of KIF3A and PCM1, another ciliogenesis gene, enhanced the sensitivity of GBM cells to the standard-of-care chemotherapeutic agent temozolomide (TMZ) [22]. Notwithstanding the potential extraciliary functions for KIF3A and IFT88, our findings suggest that GBM cilia play an important role in tumor growth and therapeutic resistance.

Other studies have suggested an anti-mitogenic role of primary cilia in GBM. U-251MG cells overexpress cell cycle-related kinase (CCRK) and display low frequencies of cilia. Knocking down CCRK in these cells increased the frequency of ciliated cells and slowed tumor proliferation, and overexpressing CCRK suppressed GBM cell ciliogenesis and promoted tumorigenesis [23]. Additionally, a recent study by Loskutov et al. [24] showed that the loss of primary cilia promoted astrocyte proliferation in a lysophosphatidic acid (LPA)-dependent manner. LPA signaling drove GBM proliferation both in vitro and in vivo, with the lysophosphatidic acid receptor 1 being accumulated in the primary cilia of both astrocytes and GBM cells. Collectively, our and other groups’ findings suggest that primary cilia in GBM could have dual and opposing effects on tumor pathogenesis, as has been shown in medulloblastoma [4, 7, 25].

Here, we extend our previous studies of GBM primary cilia by examining the L0 patient-derived cell line that we previously reported to be SHH responsive [12]. As we found that the L0 cell line and various GBM biopsies display ARL13B^+^ cilia, we live-imaged GBM cells and cilia stably expressing Arl13b:GFP. Our results demonstrate, for the first time in patient-derived cancer cell cultures, the dynamic attributes of these cilia during live cell proliferation. These characteristics include an active release of vesicles from distal ciliary tips into the tumor microenvironment, vesicles carrying material that may contribute to tumor pathogenesis. Furthermore, our findings suggest that the intracellular levels of ARL13B might impact glioma growth.

## Results

### PiggyBac transposon-mediated delivery of Arl13b:GFP into patient-derived GBM cells permits the visualization and tracking of GBM primary cilia and ciliated tumor cells

**Fig. 1:**
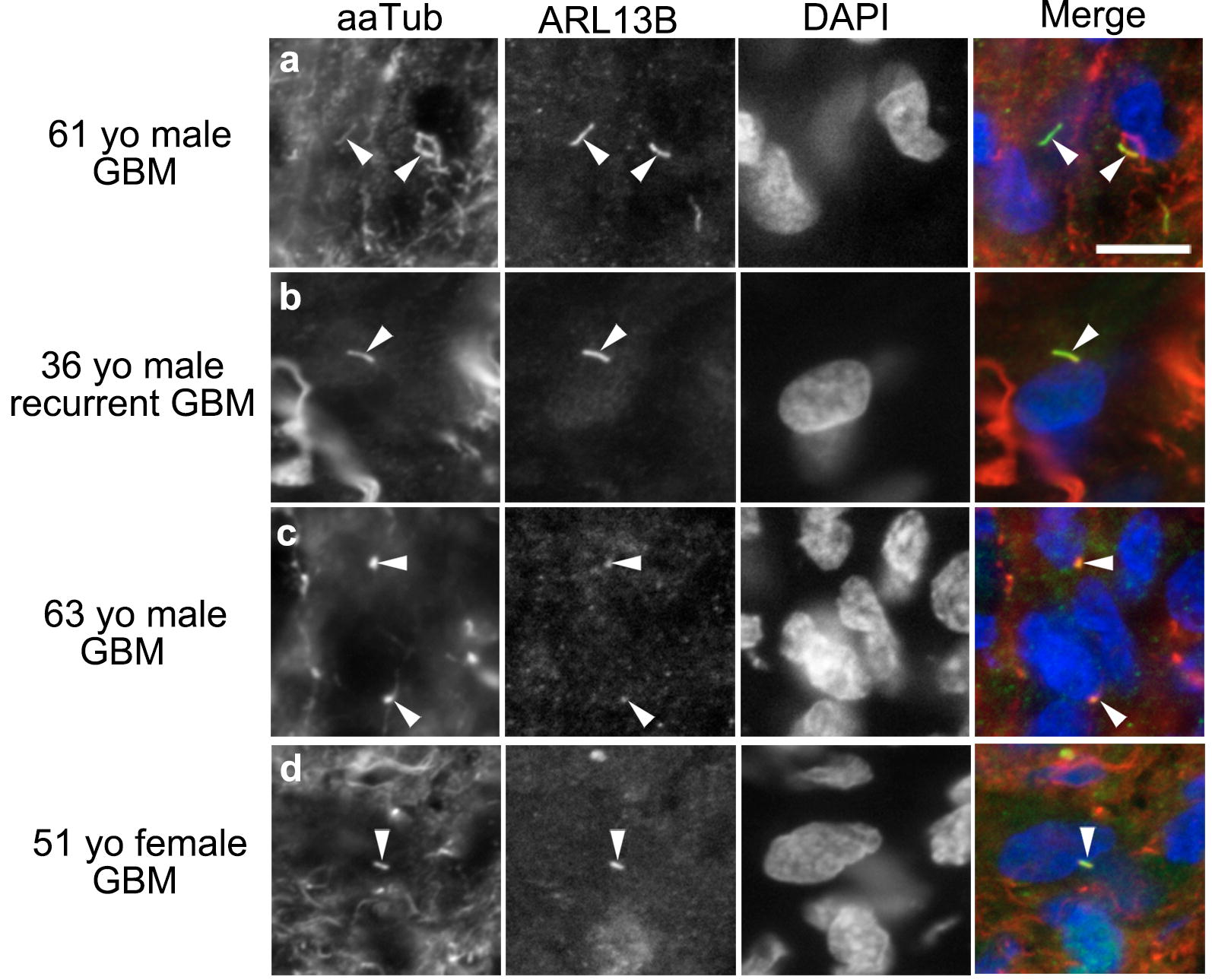
ARL13B^+^ primary cilia are present in GBM biopsies. **a**–**d** Representative confocal images of tumor biopsies from patients of the indicated age and sex. Biopsy sections were immunostained for acetylated alpha-tubulin (aaTub; first column, red), a marker of the ciliary axoneme, and ARL13B (second column, green), and co-labeled with nuclear marker DAPI (third column, blue). Arrowheads point to double-labeled cilia in each tumor section. Scale bar in **a** = 10 μm

We previously reported that cilia were detectable in patient-derived GBM cell lines and biopsies [11]. In all cell lines and patient-derived xenografts that we examined, we found that those cilia were ARL13B^+^ [11, 12]. Arl13b is a small, membrane-bound GTPase that is required for cilia formation and structure [26–29]. In the present study, we examined additional GBM biopsies and found ARL13B^+^ cilia colocalized with acetylated alpha-tubulin, a tubulin concentrated in the ciliary axoneme (Fig. 1a–d), confirming our previous findings that ARL13B is present in human GBM primary cilia.

**Fig. 2:**
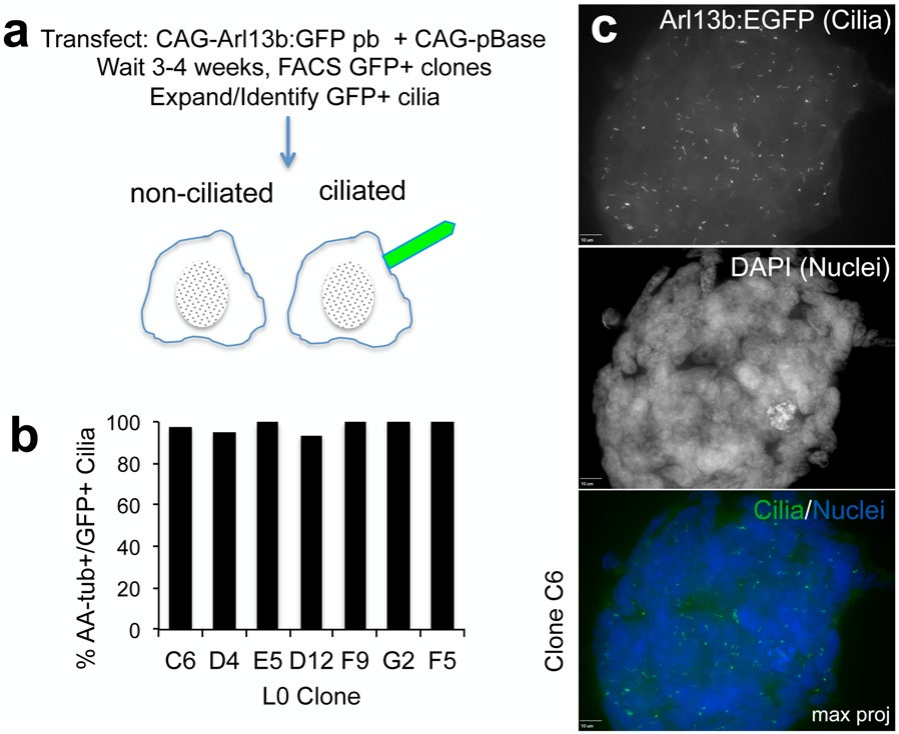
Arl13b:GFP-expressing glioma cell lines were generated using PiggyBac transgenesis. **a** PiggyBac transgenesis was used to insert GFP-tagged, full-length mouse Arl13b into the genome of GBM patient-derived cells. Three to four weeks after transfection, cells were FAC-sorted and GFP^+^ clones expanded and analyzed for the presence of GFP^+^ cilia. **b** Quantification of the percentage of acetylated alpha-tubulin^+^ (aaTub^+^) cilia that were Arl13b:GFP^+^ across seven Arl13b:GFP^+^ L0 clones. **c** Confocal maximum projection image of an unstained L0 clone C6 tumor sphere expressing Arl13b:GFP^+^ cilia (green). Nuclei were labeled with DAPI (blue). Scale bar = 10 μm

Various transgenic mouse models (e.g., expressing Arl13b:mCherry) have been employed to tag and track live ciliated cells during normal mouse embryogenesis and into adulthood [30, 31]. To visualize GBM primary cilia, we exploited our observation that ARL13B endogenously localizes to those cilia in GBM cells and used the piggyBac transposase method [32–34] to insert a C-terminal GFP-tagged full-length mouse Arl13b into the genome of patient-derived GBM cell line L0 by co-transfecting two cDNA vectors encoding pBase and CAG-Arl13b:GFP flanked by pb insertion/recognition sequences (Fig. 2a). After FAC-sorting and expanding the GFP^+^ clones, we identified several cell lines that displayed Arl13b:GFP^+^ cilia in all gliomaspheres, under fluorescence and without the need for immunostaining (Fig. 2b).

To determine if the majority of primary cilia formed by the Arl13b:GFP^+^ clones expressed GFP, we immunostained cells for aaTub and quantified the percentage of aaTub^+^ cilia that were also GFP^+^. We found that virtually all aaTub^+^ cilia were also GFP^+^ (Fig. 2c), indicating that the Arl13b:GFP transgene was efficiently passed onto and expressed in all daughter cells. The GFP^+^ cilia displayed variable morphologies, occasionally showing an enlarged distal tip (Additional file 1). Many GFP^+^ cilia were also elongated compared to wild-type (WT) primary cilia, which was likely due to the overexpression of Arl13b in those clones and its role in promoting ciliary membrane extension and elongation. It is important to note that this lengthening was not found to alter the underlying cilia ultrastructure or its ability to process SHH signaling [29].

### Time-lapse imaging of GBM cilia reveals their dynamic behavior, including ciliary tip excision

Using the L0-derived Arl13b:GFP^+^ cell lines we generated, we were able to assess the morphology and behavior of Arl13b:GFP^+^ primary cilia in live proliferating GBM cells. We observed the formation of GFP^+^ cilia shortly after mitosis (Additional file 2). This finding is consistent with our observations that cilia are typically absent from mitotic cells in the parental L0 cell line (data not shown) and with studies of mammalian cells showing that primary cilia are disassembled prior to mitosis and re-assembled in G0 or early G1 phase [35–40]. Interestingly, we noticed that some GBM cilia exhibited dynamic changes in morphology and orientation (Additional file 3). The cilia were observed to extend, retract, and re-extend. Between neighboring ciliated cells, the cilia sometimes appeared to come in very close contact with each other (Additional file 4).

**Fig. 3:**
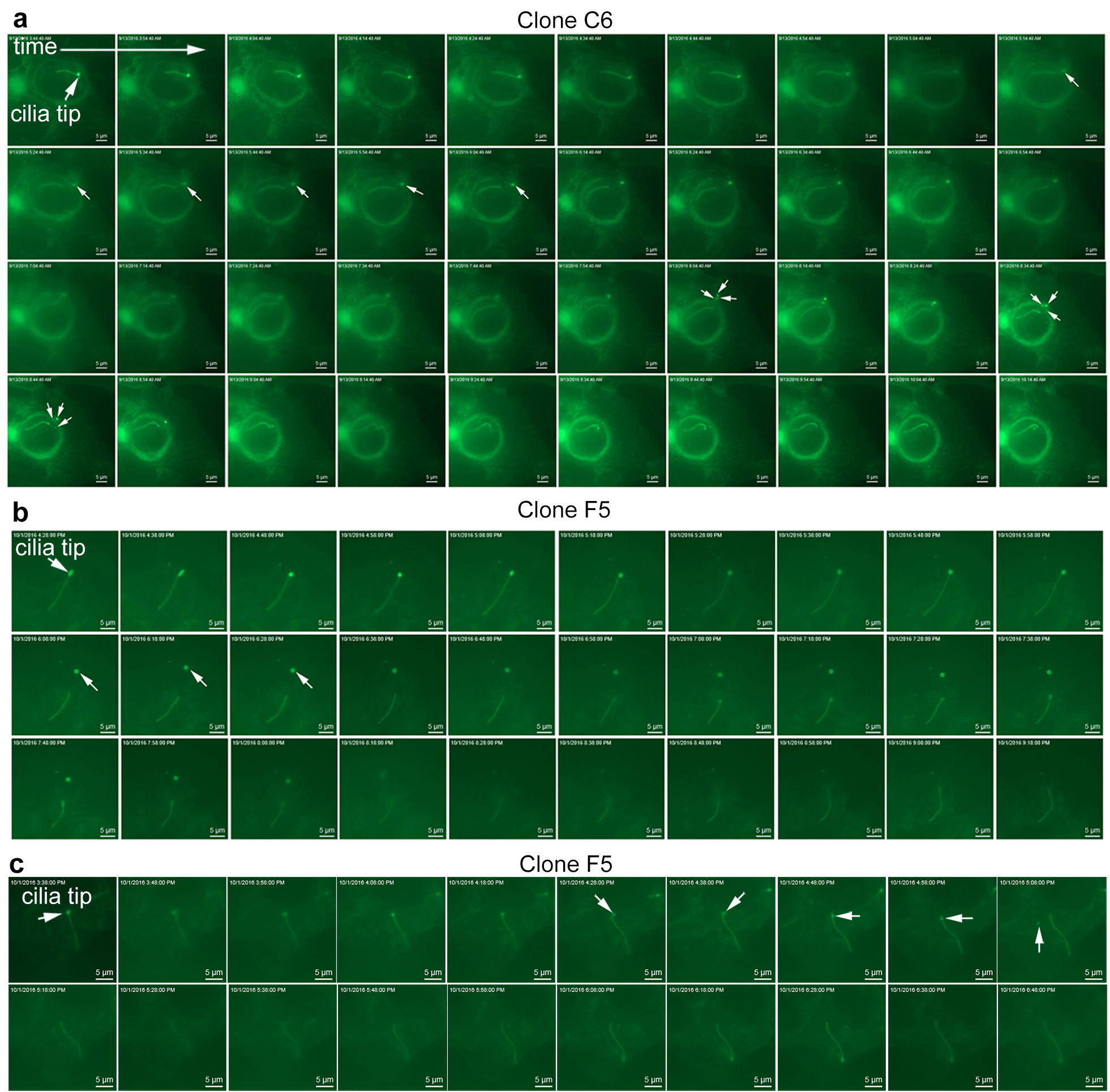
Time-lapse images and movies show ciliary tip excision in multiple L0 Arl13b:GFP^+^ clones. **a**–**c** Time-lapse image series of two different Arl13b:GFP^+^ clones (C6 and F5) in which the cilia tips appear to pinch off (arrows about halfway through each of the still images). In the top example in **a**, arrows in the third and fourth row of images show the released vesicle breaking apart and dispersing as the cell rounds up and divides, shortly after the last frame (not shown). The accompanying movie files for **a**, **b**, and **c** are shown in Additional files 5, 6, and 7, respectively

Intriguingly, we also observed the distinct pinching or budding off of ciliary tips in several GBM clones (Fig. 3, Additional files 5, 6, 7, 8 and 9). The fate of the released vesicles could not be determined because these either broke apart into smaller vesicles (e.g., Fig. 3a, Additional file 5) and/or floated away, out of the field of view. We observed one cilium that appeared to extend to nearly the length of a cell, release its ciliary tip, and then rapidly retract its axoneme (Additional file 10). The size of the excised vesicles seemed to vary. Some vesicles were small, about the diameter of a cilium, while other cilia released large vesicles from their tip. We also observed scissions at considerable distances from the cilia tips, resulting in ~ 1–5 µm-long vesicles that broke down into smaller pieces (Additional files 11, 12, 13). Thus, GBM cell primary cilia are able to display dynamic behaviors that have not been previously described on cancer cells.

### Ciliary tip excision in GBM may occur via mechanisms similar to those described in non-cancer cells

**Fig. 4:**
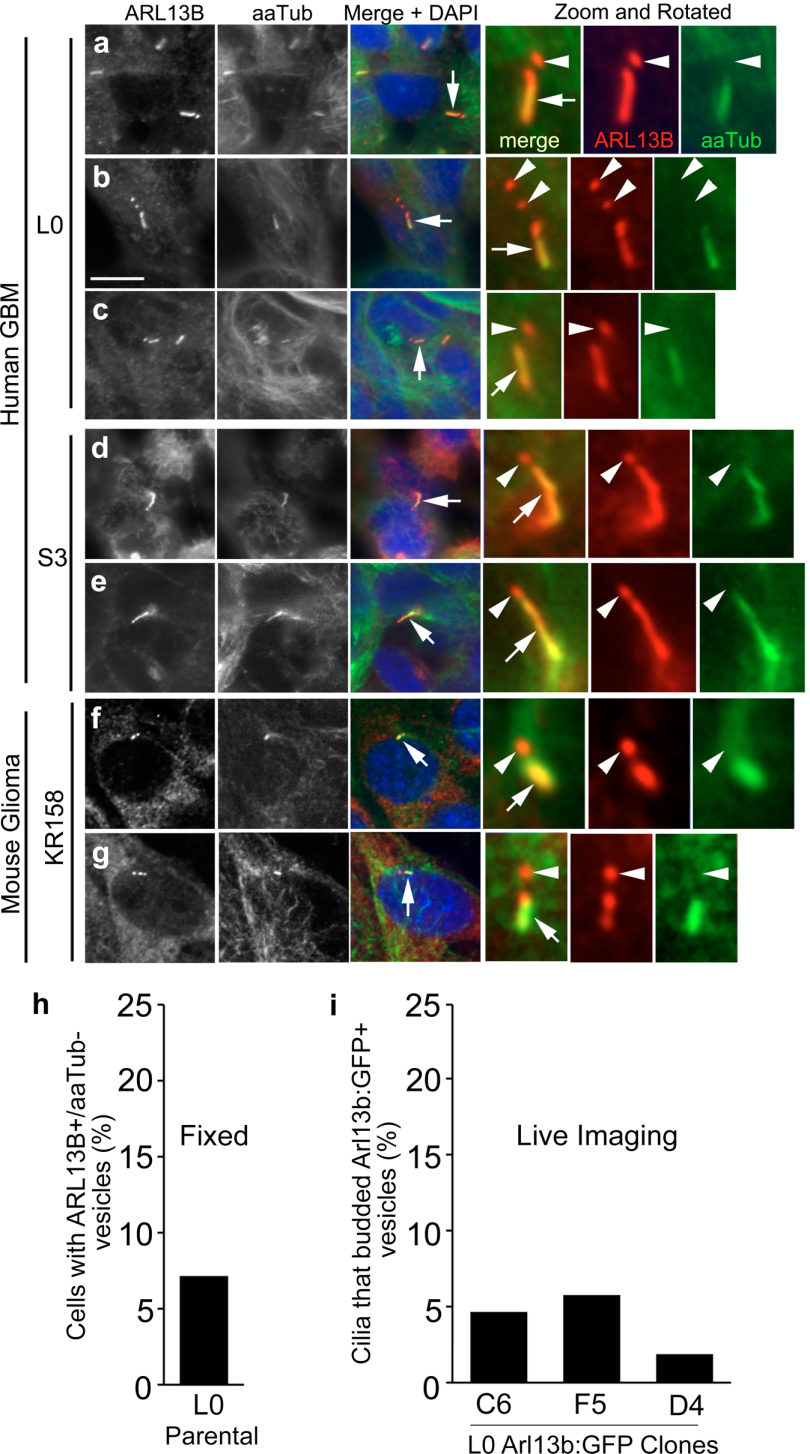
GBM cells display cilia-associated vesicles that express endogenous Arl13b but lack aaTub. Confocal images of parental human GBM L0 (**a**–**c**) and S3 (**d**, **e**) and mouse glioma KR158 cells (**f**, **g**) that were immunostained for Arl13b (red) and aaTub (green). Nuclei were stained with DAPI (blue). Cilia (arrows in the merged panel) are enlarged and rotated in the right three panels. Arl13b^+^/aaTub^−^ puncta (arrowheads) are observed adjacent to Arl13b^+^/aaTub^+^ primary cilia (arrows) in each cell line. Scale bar = 10 μm. **h** Percentage of fixed, parental L0 ciliated cells that displayed ARL13B^+^/aaTub^−^ vesicles. **i** Percentage of cilia that released Arl13b:GFP^+^ vesicles during time-lapse imaging of the 3 indicated L0 clones

Recent studies of NIH/3T3, hTERT-RPE, IMCD3, and mouse embryonic fibroblast cells found that excised cilia tips contain Arl13b but lack acetylated tubulin [41, 42]. After fixation, we immunostained several Arl13b:GFP^+^ GBM clones, GBM patient-derived L0 and S3 cell lines, and mouse KR158 glioma cells (characterization of cilia in Additional file 14) for Arl13b and aaTub. Across all parental cell lines, we observed puncta that were adjacent to the tips of Arl13b^+^/aaTub^+^ cilia and, although Arl13b:GFP^+^ or Arl13b^+^, were aaTub^−^ (Fig. 4a–g, Additional file 15). We found that 16/223 (~ 7.2%) of ciliated cells displayed ARL13B^+^/aaTub^−^ vesicles in the L0 parental cell line (Fig. 4h). Similarly, among several of the Arl13b:GFP L0 clones, we observed Arl13b:GFP^+^ vesicles released from 4/86 (~ 4.7%) cilia in clone C6, 6/104 (~ 5.8%) cilia in clone F5, and 4/206 (1.9%) cilia for clone D4 (Fig. 4i). Thus, it appears that the frequency of vesicle release occurs at a low level in both parental and Arl13b:GFP clone-derived cell lines.

**Fig. 5:**
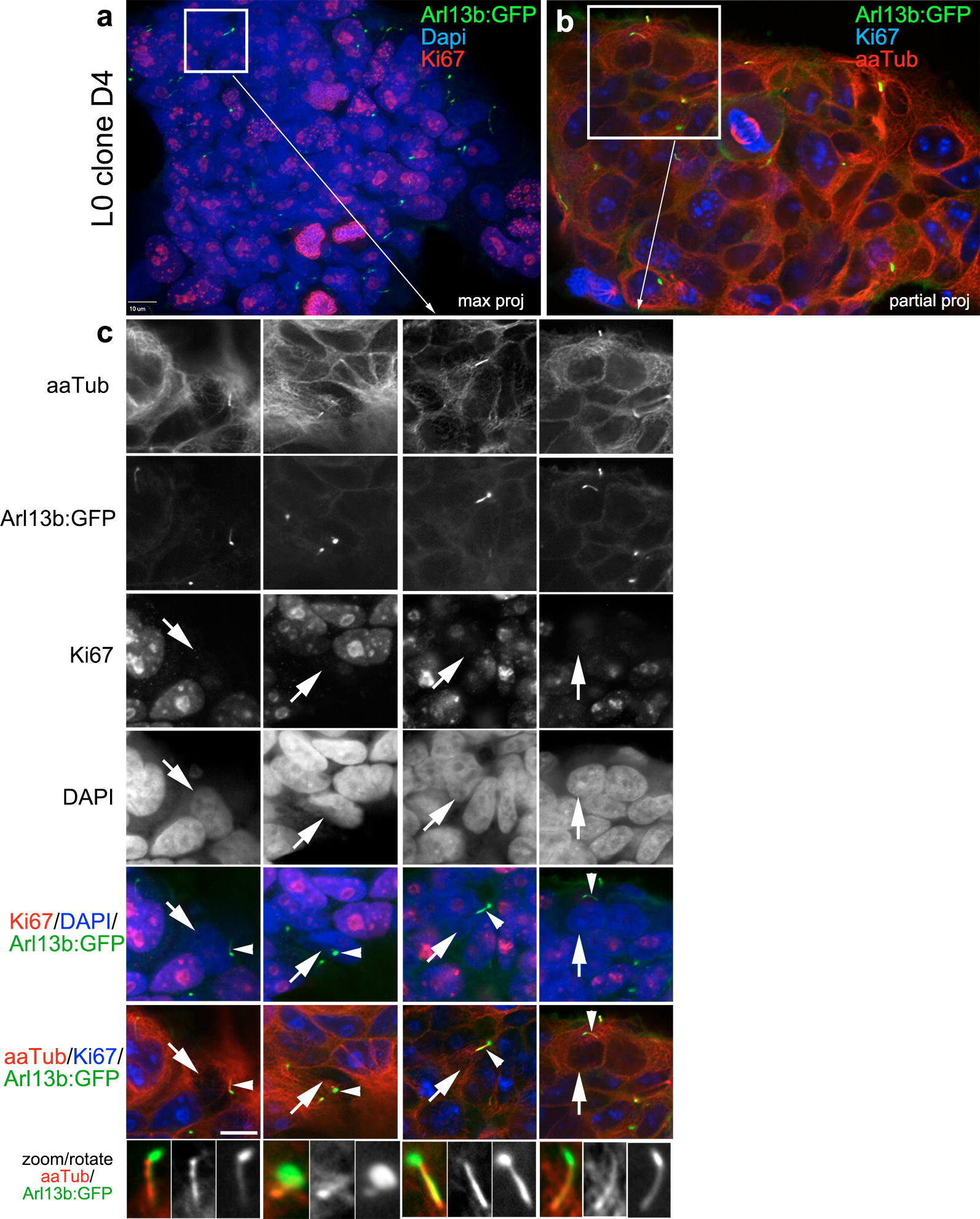
Ciliary vesicle formation occurs during the G0 phase of the GBM cell cycle. Arl13b:GFP^+^ clones were immunostained with antibodies against Ki67 (red), a marker of the active (non-G0) phases of the cell cycle, or aaTub (red), to identify cilia axonemes. Nuclei were stained with DAPI (blue). **a**, **b** Show lower magnification views of stained cells for the indicated antibodies. Boxes are enlarged in **c**. **c** Four examples (arranged in columns) of GBM primary cilia (arrowheads) that appear to have formed vesicles at their tip, on cells whose nuclei lack/display very weak levels of Ki67 (arrows). Scale bar in **a**, **c** = 10 μm

Since the release of ciliary vesicles in non-cancer cell types was reported to occur during the G0 phase and to be necessary for cell cycle progression [42], we next studied the timing of ciliary budding in relation to the cell cycle of GBM cells. We examined fixed cultures of Arl13b:GFP^+^ clones that had been immunostained with antibodies against aaTub and Ki67, a marker of the active (i.e., non-G0) phases of the cell cycle. GBM cilia that appeared to be initiating vesicle excision were associated with cells whose nuclei lacked or displayed very weak levels of Ki67 (Fig. 5).

**Fig. 6:**
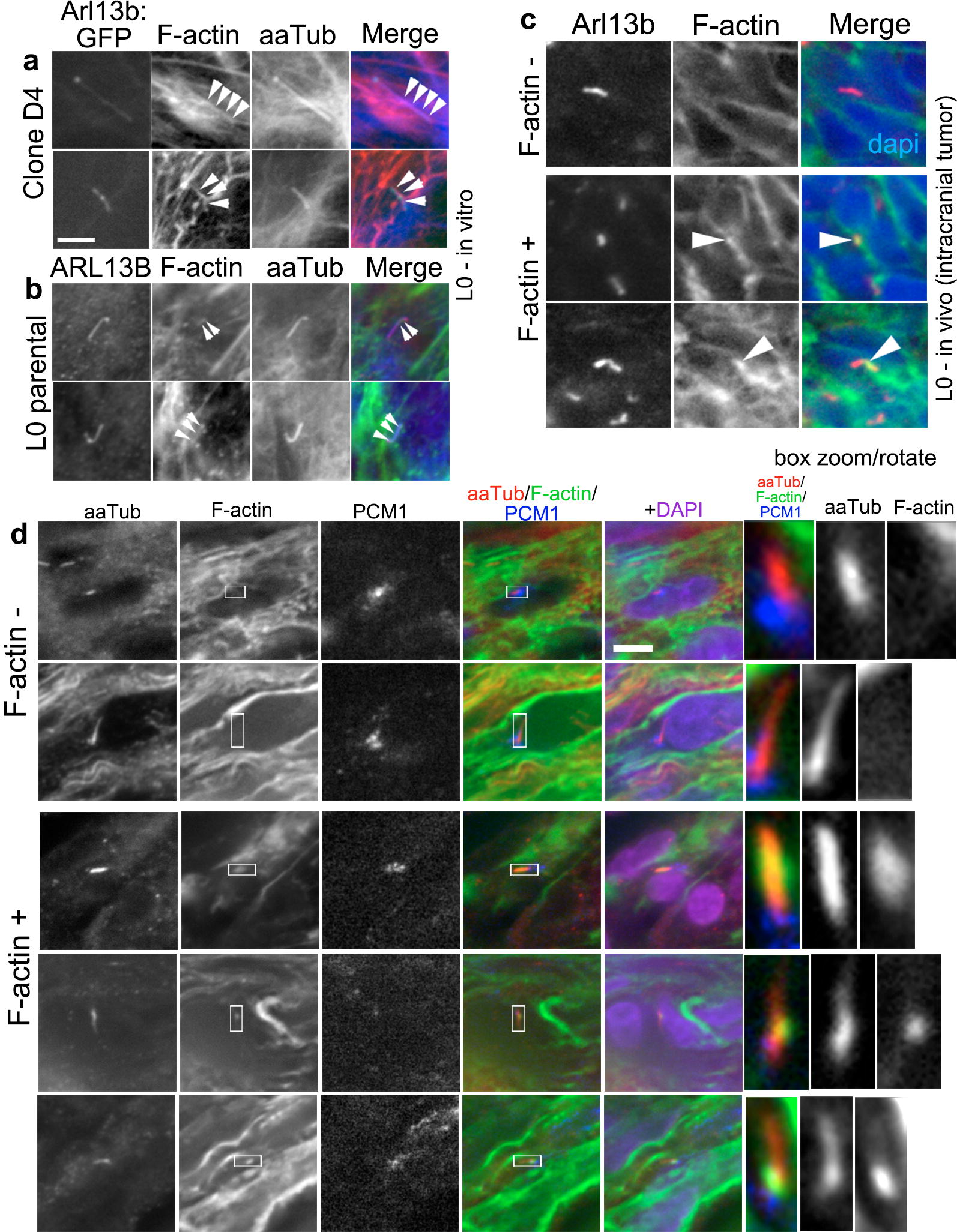
F-actin is detectable in GBM cell cilia. L0 Arl13b:GFP^+^ clones (**a**), parental L0 cells (**b**), L0 xenograft-derived tumors (**c**), and a patient biopsy (**d**) were immunostained for different cilia markers and Alexa Fluor 488-conjugated phalloidin that labels F-actin. Scattered primary cilia in which F-actin (arrowheads) colocalized with cilia markers in two examples of clone D4, parental L0 cells, L0 xenografts, and a patient biopsy are shown. Patient biopsies were stained for aaTub (red), F-actin (green), and pericentriolar material 1 (PCM1; blue), a protein that localizes to centriolar satellites around the ciliary base, with nuclei stained for DAPI (purple). Examples of F-actin-negative cilia are shown in the xenografts and biopsy. Scale bar in **a**, **d** = 5 μm

Several studies suggest that the mechanism underlying ciliary tip excision depends on the recruitment of F-actin and other actin-regulatory proteins into the cilium [41–43]. Thus, we co-immunostained GBM patient-derived cell lines, biopsies, and xenografts for ciliary markers and a fluorescently tagged phalloidin conjugate that labels F-actin. Across all groups, we found scattered cilia where F-actin was colocalized with aaTub^+^ cilia (Fig. 6). These data support the possibility that ciliary tip excisions in GBM cells may utilize an F-actin-dependent mechanism similar to the one described in non-cancerous cells.

### Conditioned media containing ciliary vesicles promotes the proliferation of ciliated GBM cells

**Fig. 7:**
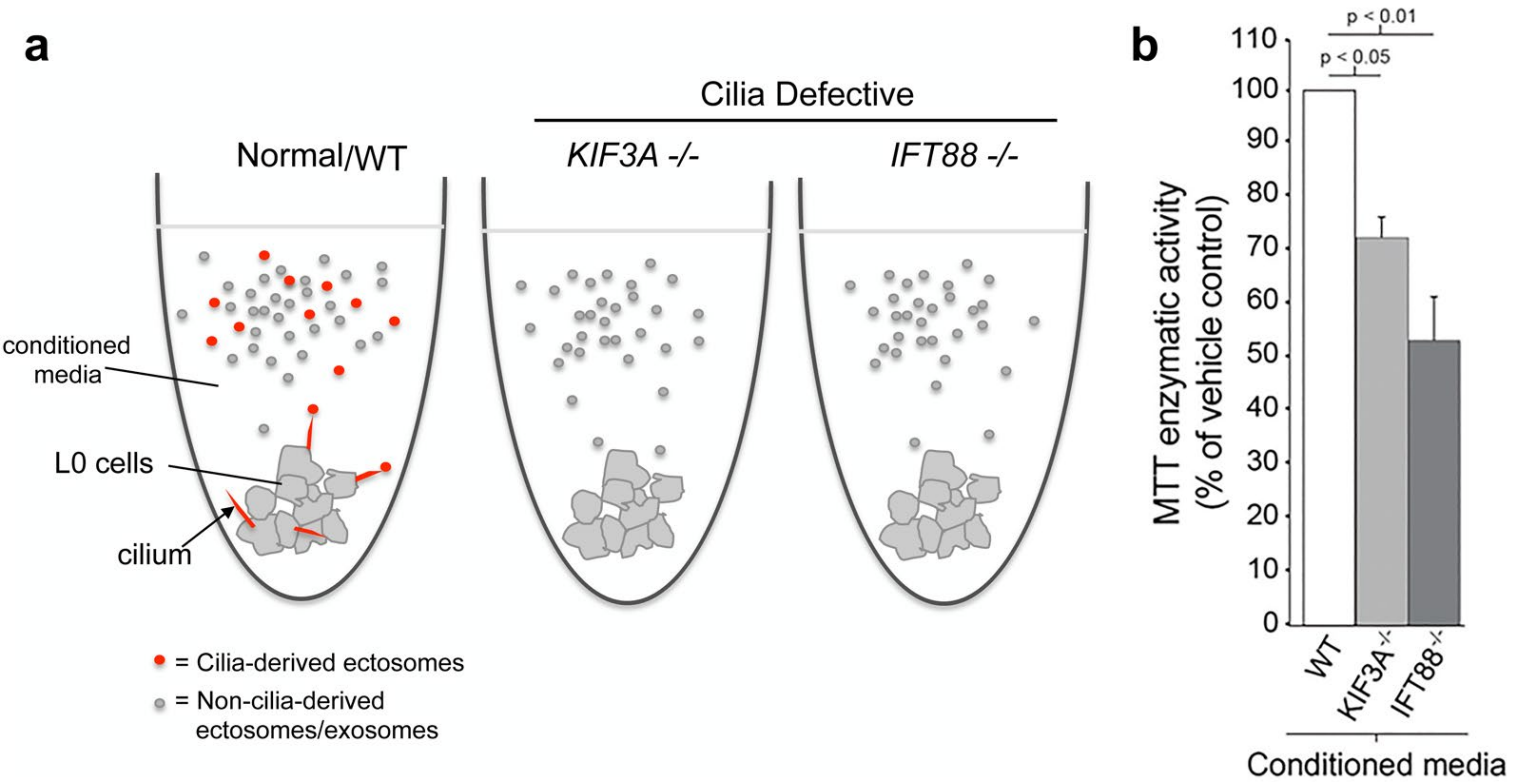
Conditioned media from ciliated GBM cell cultures promotes the proliferation of ciliated GBM cells. **a** Ciliated L0 GBM cells were exposed to purified conditioned media collected from either regular/wild-type (WT) ciliated L0 cell cultures (putatively containing cilia and extraciliary vesicles) or cilia-defective (KIF3A^−/−^ or IFT88^−/−^) L0 cell cultures (putatively containing extraciliary vesicles only). **b** Bar graph shows MTT enzymatic activity, expressed as a percent of WT control. Statistics were performed using a one-way ANOVA with Bonferroni post hoc test

Since we observed that GBM cell cilia release variably sized vesicles into their microenvironment, we next investigated whether media containing these vesicles affected GBM cell proliferation. We compared WT GBM cell conditioned media and conditioned media from cultures of cilia-depleted lines that lack KIF3A or IFT88, two proteins indispensable for ciliogenesis. These lines were generated using CRISPR/Cas9 ([22] and Additional file 16). Without IFT88, cells cannot form primary cilia and thus cannot release ciliary vesicles [42]. Using a multi-step differential ultracentrifugation process that has been shown to isolate extracellular vesicles from conditioned media [41, 42], we purified the conditioned media of WT ciliated and cilia-depleted (*KIF3A*^−/−^ or *IFT88*^−/−^) L0 cell cultures and exposed ciliated or cilia-depleted cells to either of these purified media (Fig. 7a). We found that the proliferation of ciliated GBM cells was significantly enhanced in the presence of conditioned media obtained from ciliated cell cultures when compared to conditioned media collected from cilia-depleted cell cultures, which should be depleted of ciliary vesicles (Fig. 7b).

**Fig. 8:**
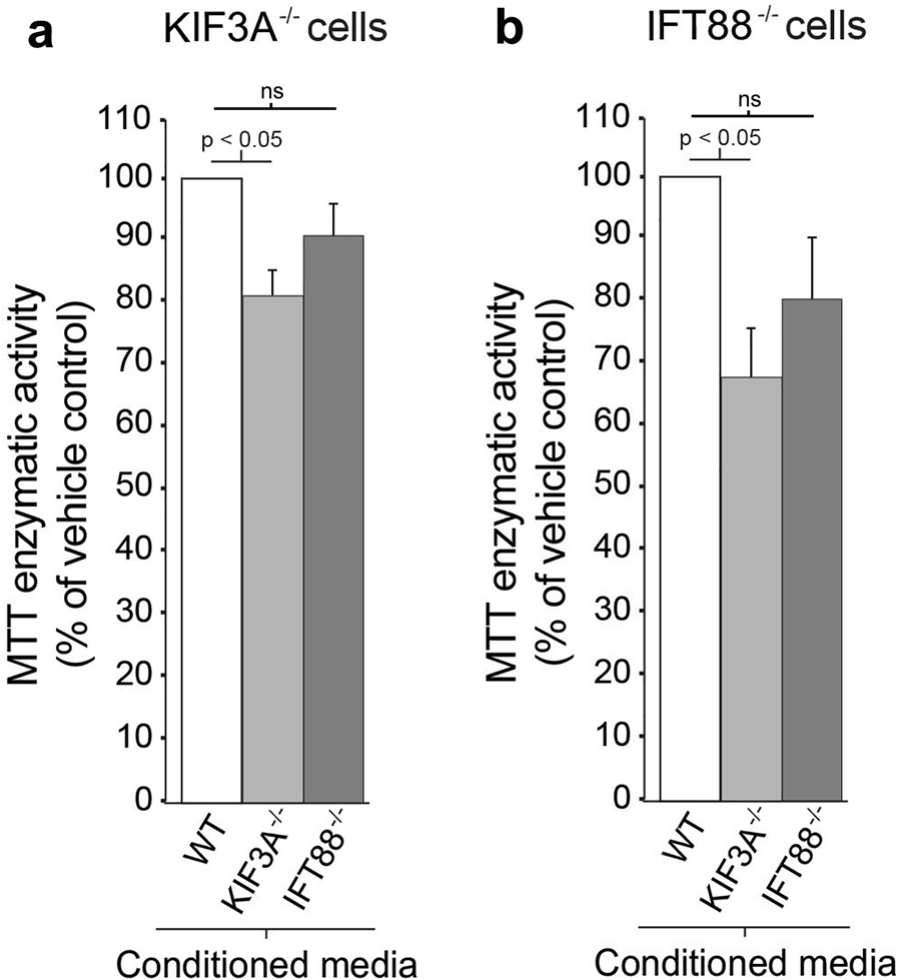
Conditioned media from ciliated GBM cell cultures does not promote the proliferation of cilia-depleted GBM cells. Cilia-depleted *KIF3A*^−/−^ (**a**) or *IFT88*^−/−^ (**b**) L0 GBM cells were exposed to purified conditioned media collected from either wild-type WT, *KIF3A*^−/−^, or *IFT88*^−/−^ L0 cell cultures. Bar graph shows MTT enzymatic activity, expressed as a percent of WT control. Statistics were performed using a one-way ANOVA with Bonferroni post hoc test

Given the molecular and cellular heterogeneity often associated with GBM, and since we found that a subset of GBM cells generate progeny that seem unable to form primary cilia [12], we then examined whether conditioned media from ciliated cells could also affect the proliferation of cilia-depleted GBM cells. We observed that conditioned media from WT ciliated and IFT88^−/−^ cilia-depleted GBM cell cultures had the same effect on the proliferation of KIF3A^−/−^ or IFT88^−/−^ cells, suggesting that ciliary vesicles do not affect the division of cilia-depleted cells (Fig. 8). Intriguingly, the proliferation of both KIF3A^−/−^ and IFT88^−/−^ GBM cells was decreased when these cells were exposed to conditioned media from KIF3A^−/−^ GBM cell cultures when compared with conditioned media from WT ciliated GBM cell cultures. Since no decrease in cell proliferation was observed when these cells were exposed to conditioned media from IFT88^−/−^ cilia-depleted GBM cell cultures, these results suggest that extraciliary factors/vesicles might affect cilia-depleted cell proliferation and that KIF3A might be involved in these effects. Collectively, our data suggest that GBM cell cilia release bioactive vesicles containing factors which promote the proliferation of ciliated tumor cell populations but have no effect on cilia-depleted cells. However, future studies are needed to definitively isolate and concentrate GBM vesicles, in order to distinguish the effects of ciliary versus extraciliary vesicles on the mitogenic potential of GBM cells.

### Arl13b overexpression in glioma cells induces ciliary smoothened enrichment and promotes GBM cell proliferation

**Fig. 9:**
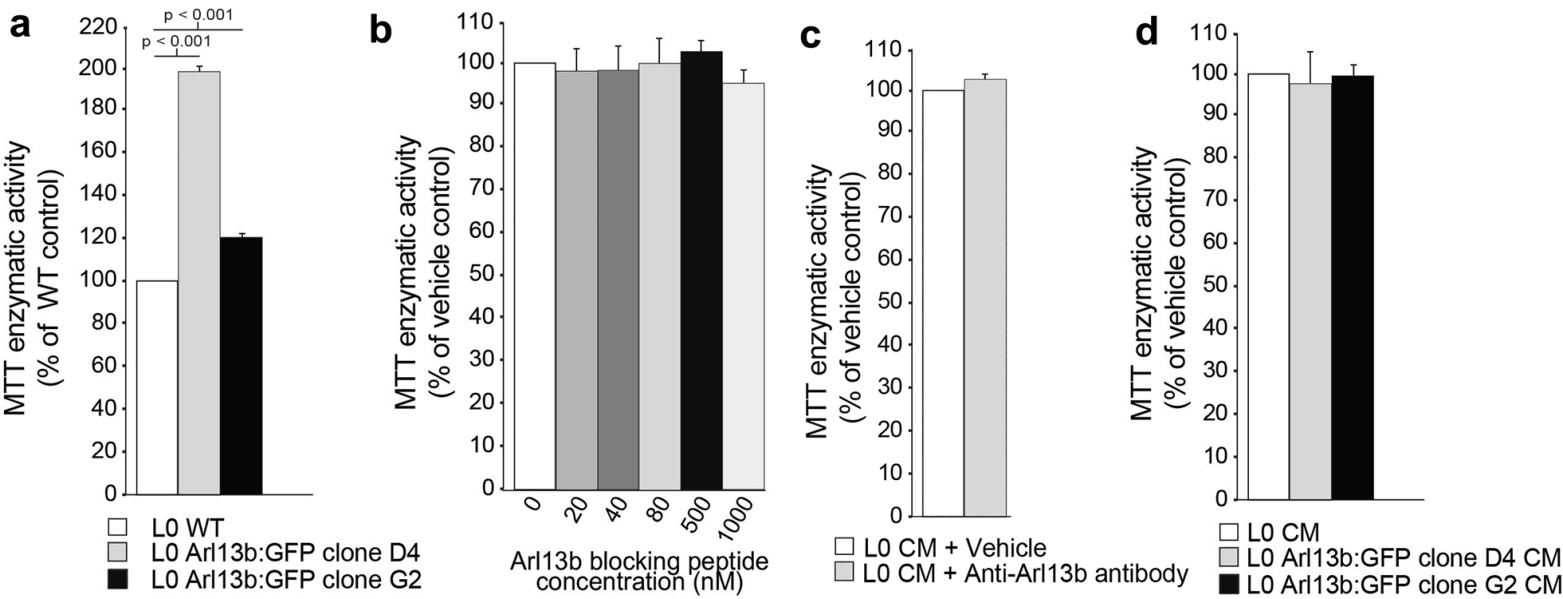
Increased proliferation of Arl13b:GFP^+^ clones is not due to extracellular Arl13b signaling. **a** MTT assays were used to compare the cell viabilities of parental L0 WT cells and Arl13b:GFP^+^ clones D4 and G2. Bar graph shows MTT enzymatic activity, expressed as a percent of WT control. Statistics were performed using a Student’s *t* test. **b**, **c** Parental L0 GBM cells were exposed to ciliated culture-derived purified conditioned media to which was added a range of concentrations of an Arl13b blocking peptide (**b**) or an anti-Arl13b antibody [0.041 μg/μl] (**c**). Bar graphs show MTT enzymatic activity, expressed as a percent of control. Statistics were performed using a one-way ANOVA with Bonferroni post hoc test. **d** Parental WT L0 cells were exposed to purified conditioned media collected from either Arl13b:GFP^+^ D4 and D2 clone cultures or wild-type (WT) L0 cell cultures. Bar graph shows MTT enzymatic activity expressed as a percent of WT control. Statistics were performed using a Student’s *t* test

Arl13b has been recently implicated in promoting both gastric tumor growth [44] and medulloblastoma [45]. The Arl13b:GFP^+^ clones, whose cilia were observed to release Arl13b^+^ vesicles, were more proliferative than the WT parental cell line (Fig. 9a). Thus, we next investigated whether Arl13b associated with those extracellular ciliary vesicles promoted the proliferation of GBM cells. We reasoned that GBM ciliary vesicles may contain membrane trafficking proteins, such as ARL13B, acquired from the ciliary plasma membrane [41, 46–48], and that incubating conditioned media with small blocking peptides or antibodies could potentially bind to those membrane proteins and disrupt Arl13b’s function. However, we found that the addition of a range of concentrations of an Arl13b blocking peptide (Fig. 9b) or an anti-Arl13b antibody (Fig. 9c) to WT conditioned media did not alter the proliferation of GBM cells exposed to these media when compared with untreated WT media. We also examined whether the purified conditioned media from Arl13b:GFP^+^ clones, which we observed contained high levels of Arl13b:GFP by WB (data not shown), stimulated GBM cell proliferation. We found that these media did not stimulate tumor cell proliferation more than WT media (Fig. 9d). These findings suggest that the increased proliferation of the Arl13b:GFP^+^ clones is not due to the Arl13b carried by the vesicles released by their cilia but might result from the enhanced intracellular expression of Arl13b in those clones.

**Fig. 10:**
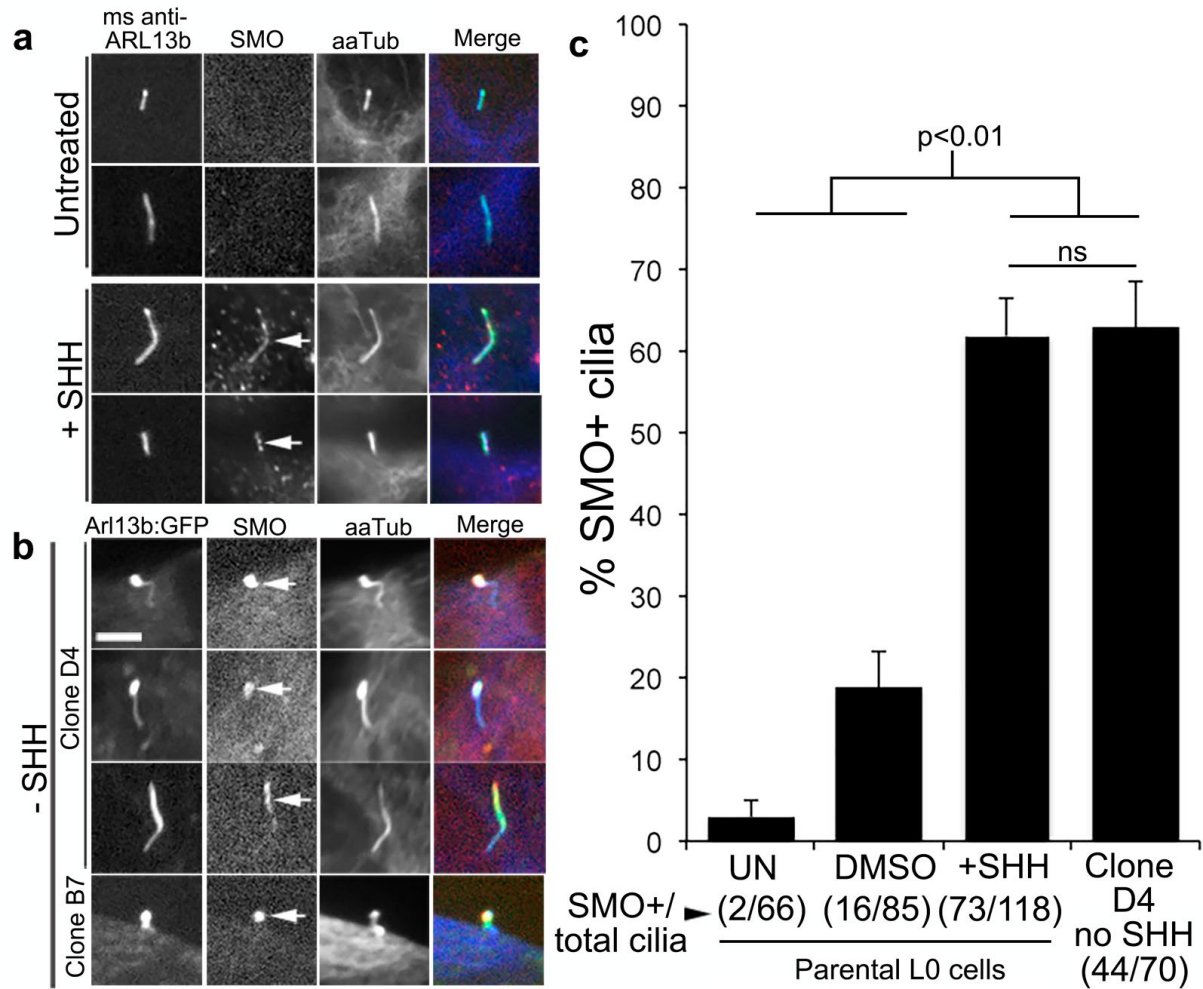
Arl13b overexpression leads to abnormal SMO enrichment in GBM cell cilia. Confocal images of **a** cultured L0 cells untreated or exposed to SHH [1 μg/ml] and immunostained for ARL13B (green), SMO (red), and aaTub (blue) and **b** Arl13b:GFP^+^ clones co-stained for SMO (red) and aaTub (blue). L0 GBM cells that had been exposed to SHH and Arl13b:GFP^+^ clones displayed SMO (arrows) that was colocalized with Arl13b^+^/aaTub^+^ cilia. **c** Percentage of cells with SMO^+^ cilia in untreated, DMSO-treated, SHH-treated L0 wild-type (WT) cells, and Arl13b:GFP^+^ clone D4

Ciliary Arl13b is important for mediating signaling in response to SHH [26, 28, 49]. A recent study found that Arl13b directly binds to and stabilizes SMO and that higher levels of Arl13b prevent the degradation of SMO and accelerate tumor growth [44]. Thus, we investigated whether the overexpression of Arl13b in our Arl13b:GFP^+^ clones affected SMO expression or ciliary localization. Confirming our previous findings, SMO was largely undetectable in the cilia of untreated parental L0 cells but readily visualized in the primary cilia of SHH-treated L0 cells (Fig. 10a). In contrast, we observed a robust enrichment of SMO in Arl13:GFP^+^ clone cilia in the absence of SHH (Fig. 10b). Quantification of the data showed that the percentage of cells with SMO^+^ cilia was significantly increased in Arl13b:GFP^+^ clones compared to WT GBM cells and was similar to the percentage of SMO^+^ cilia in SHH-treated parental L0 cells (data for clone D4 shown; Fig. 10c). Collectively, these data suggest that the overexpression of intracellular Arl13b in the Arl13b:GFP^+^ clones may promote an enrichment of SMO in their cilia and, consequently, a stimulation of cell proliferation. It is noteworthy that our analysis of the TCGA database revealed that higher expression levels of *ARL13B* and *SMO* in low-grade glioma correlate with shorter overall patient survival (Additional file 17). Whether the increase in intracellular Arl13b and ciliary SMO stimulates the release of ciliary vesicles, further promoting the proliferation of GBM cells, requires further investigation.

## Discussion

Here, we describe a potential new mode of intercellular communication in glioma that originates from the tumor cells’ primary cilium. Our data suggest that glioma primary cilia may release bioactive material capable of promoting tumor cell proliferation into the tumor microenvironment. Although other groups have shown that glioma cells release extracellular vesicles carrying oncogenic material [50], the ciliary vesicles we describe could constitute a new subclass of tumor-derived extracellular vesicles that serve to propagate the cancer.

Our results may be similar to what was observed in various pharmacologically drug-resistant human cancer cell lines (e.g., HCCC4006 lung adenocarcinoma, A549 lung carcinoma, rhabdoid tumor A204 and Dasatinib-resistant subline DasR), in which apparent ciliary tip fragmentation was described [51]. However, those studies found that the fragment tips contained both Arl13b and aaTub, whereas we and others have found that the Arl13b^+^ vesicles are weak or negative for aaTub [42, 47]. In non-cancerous cells, primary cilia have been shown to release ectosomes from their tips into the extracellular milieu [41, 42, 47, 52–54]. These vesicles were reported to contain matrix metalloproteinases, G-protein-coupled receptors, transcription factors, and signaling mediators such as Arl13b. The release occurs mostly during the G0/quiescent cell cycle phase and is required for cilia disassembly and cell cycle re-entry (e.g., [42]). The fact that the majority of glioma cells in our cultures were actively dividing may explain the low frequency of ciliary tip excisions that we were able to observe during the live imaging sessions and in fixed cultures. Since we did not simultaneously monitor cell cycle phase markers along with our tagged cilia, we cannot affirm whether the vesicle release from cilia occurs during G0 or other phases of the cell cycle. We did observe cilia that appeared to be in the process of budding were extending from cells lacking Ki67, which suggests that this phenomenon might occur in quiescent glioma cells, similarly to non-cancerous cells [42]. If the process of ciliary vesicle release does drive glioma cell cycle progression and occurs during quiescence—when cells are most resistant to conventional therapies—it is tempting to speculate that targeting this process could yield novel therapeutic strategies that would inhibit tumor progression and currently inevitable recurrence.

We next examined the mechanisms that might contribute to glioma cell cilia excision. Serum exposure has been reported to significantly elevate the levels of Arl13b and other ciliary components in conditioned media [42], and we observed the release of glioma cilia vesicles containing Arl13b:GFP in the presence of 5% serum containing known cellular growth factors. Therefore, growth stimulation might be promoting cilia vesicle release in glioma cells. In addition, Nager et al. [41] have reported that the application of the SMO agonist SAG increases the concentration of GPCRs at the cilia tips as well as their ectocytosis. Thus, the increase in SMO associated with Arl13b:GFP overexpression in the GBM clones, enrichment which was particularly prevalent in the ciliary tips, may constitute one of the mechanisms stimulating the release of ciliary vesicles in glioma cells. Furthermore, the excision process requires the unexpected recruitment of F-actin and actin regulators in non-cancerous cells [41]. Interestingly, we observed cilia expressing F-actin in glioma cell cultures, xenografted tumors, and patient biopsies, at a frequency similar to the low frequency of cilia vesicles observed in those tumors. These findings raise the possibility that, like in non-cancerous cells, F-actin-based mechanisms may mediate the release of ciliary vesicles in glioma tumors; however, whether the timing and recruitment of actin regulators are similar in glioma cells requires additional characterization. Even though growth stimulation, Arl13b-mediated SMO upregulation, and F-actin may contribute to glioma ciliary excision, further studies are needed to elucidate the exact mechanisms involved in this process.

Notably, we found that purified conditioned media derived from WT GBM cell cultures, and which putatively contained ciliary vesicles, promoted the proliferation of ciliated GBM cells when compared to media from two different types of cilia-depleted GBM cell cultures. It is unclear whether the factors carried by these vesicles promoted overall tumor cell proliferation by stimulating cell division or by promoting cell survival, but our findings suggest that these ciliary vesicles may be bioactive and potentially play an important role in promoting tumor growth. We also found that the proliferation of GBM cells which were unable to form cilia was not stimulated by WT media containing ciliary vesicles, suggesting that these vesicles may affect cell proliferation through cilia-dependent mechanisms. From our live cell imaging studies, we were not able to determine the fate of these ciliary vesicles or whether they affect tumor cells in an autocrine or paracrine fashion as the vesicles often floated away, disappearing from the field of view, or broke apart after being released. It seems likely, however, that the distribution of those vesicles post-excision would be restricted to neighboring cells in the tumor environment given the density of cells in GBM and presence of the blood–brain barrier.

It should be noted that there were differences in the way we prepared our conditioned media compared to recent studies. Our experiments used conditioned media after a 40,000×*g* ultracentrifugation step whereas other groups purified vesicles with an additional ultracentrifugation step of 100,000×*g* (e.g., [41, 42]). We did not pursue higher speed centrifugation partly because the particles we observed in our time-lapse imaging varied from ~ 200 nm to over 1 μm in size, larger than the ciliary vesicles measured by others that averaged around 100 nm in diameter [41]. This size discrepancy is likely a result of the previously described role of transgenic Arl13b:GFP in stimulating ciliary membrane biogenesis [29], but we cannot rule out that other defects in the ciliary tip excision process are present in our GBM cell lines. Nevertheless, an important future experiment to pursue will be to isolate the vesicles from ciliated and cilia-depleted cells and test the mitogenic capacity of these vesicles. A technical challenge will be to separate ciliary from extraciliary vesicles in order to rule out non-ciliary factors and metabolites that might precipitate with ciliary vesicles.

Tagging GBM primary cilia with Arl13b:GFP allowed us to observe other behaviors and characteristics of those cilia and associated tumor cells. GBM cilia appeared quite dynamic, undergoing extension and retraction and displaying dynamic reorientation in our live cell cultures. Such observations have previously been made regarding migrating interneuron primary cilia [55]. As interneurons traverse through the developing brain to invade the cortical layers, they pause along their migratory paths and extend/retract their cilia, using them to probe their surrounding environment and possibly sensing and being guided by SHH signals within the cortex [56]. We also observed some GBM cells whose cilia were transiently brought in close apposition, raising the possibility that signaling could occur between GBM cell cilia. In mouse retina and liver tissue, cilia–cilia contacts have been reported and were mediated by N-linked glycoproteins and ‘melted’ prior to mitosis [57]. Although primary cilia are putatively non-motile in the brain, it is tempting to speculate that the dynamic behavior of GBM cilia allows them to actively survey their surrounding environment, thus enhancing their sensitivity to local extracellular cues and supporting the tumor cells’ proliferation and/or migration within the brain.

Overexpressing Arl13b in GBM cells also led to some intriguing changes in the SHH signaling pathway and cell proliferation. Most Arl13b:GFP^+^ L0 clones displayed SMO in their cilia as well as increased cell proliferation, two features that we previously found to only occur after SHH exposure in the WT parental cell line [12]. The enrichment of SMO in the cilia of Arl13b:GFP^+^ clones may have been due to increased Arl13b binding, which prevents SMO degradation [44]. The increased Arl13b:GFP^+^ cell proliferation appeared independent of the Arl13b carried by released ciliary vesicles. These findings suggest a novel intracellular/intraciliary interaction between Arl13b and SMO that may play a role in GBM pathogenesis and whose mechanism will need to be investigated. It is possible that even in the absence of the SHH ligand, glioma cells that fail to properly maintain specific intracellular levels of Arl13b may undergo abnormal activation of ciliary signaling through the upregulation of SMO into their cilium. Factors that regulate SMO levels, like Arl13b, may be important in tumor progression, as upregulated SMO expression is associated with decreased overall survival of GBM patients [58]. However, the loss of Arl13b has also been shown to lead to increased SMO levels in the primary cilia of non-cancer cells [28], to ultrastructural defects in the cilium [26], and to aberrant polarity and proliferation of neural stem cells in the developing brain [59]. Therefore, Arl13b may play a dual role as a key regulator of SMO trafficking into the cilium. Dysregulated levels of Arl13b in glioma cells may have significant consequences on the SHH signaling pathway and tumor cell proliferation. A recent study has shown that the disruption of Arl13b inhibits cilia-dependent oncogenic Shh overactivation in medulloblastoma [45]. Whether SMO is released in ciliary vesicles and whether this release impacts tumor cell proliferation will require further study. Furthermore, the factors promoting glioma cilia excision, as well as the ciliary vesicle contents and extracellular targets, will need further characterization.

## Materials and methods

### Cell lines and piggyBac transposon-mediated generation of Arl13b:GFP-expressing GBM cell lines

Two GBM patient-derived cell lines [line 0 (L0) (43 yo male) and SN186 (S3) (75 yo male)] were cultured as previously described [11, 12, 22]. L0 cells depleted of KIF3A using CRISPR/Cas9 were derived in previous study [22], and L0 cells depleted of IFT88 using CRISPR/Cas9 were generated using the same method (Additional file 16). The mouse KR158 glioma cells, an immortalized cell line derived from a murine grade III anaplastic astrocytoma [60], were a gift from J. Harrison. L0 and S3 cells were grown as floating spheres and maintained in DMEM/F12 medium supplemented with 2% B27, 1% penicillin–streptomycin, 20 ng/ml human EGF, and 10 ng/ml human bFGF. DMEM/F12 medium, B27 (cat # 17504-044), EGF (cat # PHG0311), bFGF (cat # PHG0026), and antibiotics (cat# 15240-062) were obtained from Gibco (Life Technologies, CA). Cell cultures were maintained in a humidified incubator at 37 °C with 5% CO_2_. When the spheres reached approximately 150 μm in diameter, they were enzymatically dissociated by digestion with Accumax (cat # AM-105; Innovative Cell Technologies, Inc.) for 10 min at 37 °C. Cells were washed, counted using Trypan blue to exclude dead cells, and re-plated in fresh medium supplemented with hEGF and bFGF. For cells grown on glass coverslips, DMEM/F12 medium was supplemented with 5% heat inactivated fetal bovine serum (FBS; cat # S11150H, Atlanta Biologicals). For SHH experiments, cells were treated with recombinant human SHH (1 μg/ml; R&D Systems) dissolved in sterile PBS and fixed 24 h later.

To generate clones that stably express Arl13b:GFP, L0 cells were grown on coverglass in 24-well plates and transfected at 60–70% confluence with a total of 500 ng per well of pCAG-pBase and pCAG-Arl13b:GFPpb vectors using 2 μl of Lipofectamine 2000 (cat # 11668-019; Life Technologies). To generate the pCAG-Ar13b:GFPpb vector, we subcloned the C-terminal GFP-tagged Arl13b sequence from pDest-Arl13b:GFP (gift from T. Caspary) into the pCAG-pb vector. After 3–4 weeks, individual GFP^+^ clones were sorted into 96-well plates each containing 250 μl of DMEM/F12 medium supplemented with hEGF and bFGF using a BD FACS Aria II Cell Sorter (BD Biosciences, San Jose, CA). Cell debris was excluded from the analysis by forward- and side-scatter gating. Subsequently, expanded clones that displayed GFP^+^ cilia under fluorescence were propagated for live imaging analyses. For transient transfections, cells were transfected at 50% confluency with pDest-Arl13b:GFP.

### Xenografts and glioma biopsies

Tumor sections derived from intracranial L0 xenografts in mice, whose tumors cells all expressed mCherry [12], were stained and analyzed. The GBM patient biopsies used in this study were harvested, de-identified, and banked by the Florida Center for Brain Tumor Research in adherence with institutional IRB guidelines. Six biopsies were used; four biopsies were histopathologically classified as Grade 4 GBM, one as recurrent Grade 2/3, and one as Grade 3. Within 1–2 h of surgical resection, biopsies were fixed in 4% paraformaldehyde (PFA; cat # 00380; Polysciences, Inc.) overnight, washed in PBS, immersed overnight at 4 °C in 30% sucrose in PBS followed by an overnight immersion at 4 °C in a 1:1 mixture of 30% sucrose/PBS and Tissue-Plus™ optimal cutting temperature compound (OCT; cat # 23-730-571; Fisher Healthcare) overnight, and frozen over liquid nitrogen in OCT. Sections (10–20 µm thick) were cut using a cryostat and placed directly onto Superfrost™ Plus coated slides (cat # 12-550-15; Fisher Scientific) and stored at − 20 °C until stained.

### Time-lapse imaging

All imaging was performed on an inverted Zeiss AxioObserver D1 microscope using a Zeiss 40×/0.95 plan Apochromat air objective. Individual 35 mm time-lapse imaging dishes (part # P35G-0-14-C; MatTek Corporation), with a glass bottom on which adherent cells had been grown to 75–95% confluence, were secured onto a stage-top incubation system and maintained in a humid chamber at 37 °C and 5% CO_2_ using a Tokai Hit System. In some experiments, nuclei were labeled and simultaneously imaged with the 5 µM DRAQ5 fluorescent probe (cat # 62254; Thermo Fisher Scientific), which was added 1 h before imaging. Using an XCite epifluorescent lamp, images of fluorescent signal were collected every 5–10 min, typically overnight, with exposure times ranging from 80 to 2500 ms (EGFP) and 20–40 ms (DRAQ5) per image depending on the clone or cell line being studied. Image acquisition and processing were performed using the Zeiss ZEN software. Movies were exported as .avi or .mov files at 5 frames per second.

### Antibodies, immunostaining and confocal analysis

Primary antibodies used for immunocytochemistry (ICC) or immunohistochemistry (IHC) included mouse anti-acetylated alpha-tubulin [1:3000 (ICC/IHC); Sigma (cat # T6793; lot # 088K4829)], mouse anti-Arl13b [1:1000 (ICC); clone N295B/66; NeuroMab], rabbit anti-Arl13b [1:3000 (IHC); Proteintech (cat # 17711-1-AP; lot # 00017960)], rabbit anti-Ki67 [1:200 (ICC); Vector (cat # VP-RM04; lot # V0523)], rabbit anti-SMO [1:1000 (ICC); Abcam (cat #ab38686; lot #GR198520-1)], and rabbit anti-PCM1 [1:1000 (ICC/IHC); Bethyl Laboratories (cat # A301-150A; lot # A301-150A-1)]. Cells/tissue were blocked in 1× phosphate-buffered saline (PBS) containing 5% normal donkey serum (NDS; cat # 017-000-121; Jackson Immunoresearch) and 0.2% Triton-X 100 for 30 min (ICC) or 1 h (IHC) at RT. Cells/tissue were incubated in primary antibodies diluted in PBS containing 2.5% NDS and 0.1% Triton-X-100 overnight at 4 °C. Cells/tissues were washed 4 times with PBS and incubated for 1 h at RT with species-specific secondary antibodies conjugated with fluorescent tags [1:400 (ICC/IHC); Jackson Immunoresearch]. To label F-actin, Alexa Fluor 488 (cat # A12379; lot # 44507A) and/or Alexa Fluor 568 (cat # A12380; lot # 41C1-1)-conjugated phalloidin (1:100; Invitrogen) were used during the secondary antibody step. Stained sections and cells were rinsed several times in PBS and coverslipped with Prolong Gold antifade media containing DAPI (cat # P36935; Life Technologies).

Confocal analysis of sections and cells was performed using an Olympus IX81-DSU confocal microscope fitted with a 60×/1.20 UPlanApo water objective. All images were captured as z-stacks (0.5 µm steps). In most cases, partial projections (2–3 planes from one z-stack) are shown.

### Cell viability assay

To assess the effects of different conditioned media on cell viability, each experimental group was plated in 96-well cell culture plates (8–12 wells per group with 10,000 cells re-suspended in 100 µl of the indicated conditioned media in each well). After 7 days, cell viability was assessed using the 3-(4,5-dimethylthiazole-yl)-2, 5-diphenyl tetrazolium bromide (MTT) (cat # M2128; Sigma) assay as previously described [12, 61]. The amount of viable cells was determined through optical density measurements (by measuring absorbance at 570 nm using an iMark™ Microplate Absorbance Reader). Experiments using ARL13B blocking peptide were prepared in sterile PBS according to the manufacturer’s instructions (cat # 33R-8244; Fitzgerald Industries International). Experiments using conditioned media pre-incubated with anti-Arl13b antibody (cat # 17711-1-AP; lot # 00017960; Proteintech) was prepared by diluting the antibody 1:1000 [0.041 μg/μl].

### Cell culture conditioned media purification

Cells were plated at 250,000 cells per T25 flask in 10 ml of media (DMEM/F12 medium supplemented with 2% B27, 1% penicillin–streptomycin, 20 ng/ml human EGF, and 10 ng/ml human bFGF). To generate conditioned media containing the ciliary vesicles and to remove cell debris, the media from each cell culture group were collected after 7 days in culture and purified using a multi-step differential centrifugation process (all steps at 4 °C), which has been shown to isolate extracellular vesicles [41, 42]. The media were collected and first spun at 600*g* for 5 min. The resulting supernatant was then spun at 2000*g* for 20 min, followed by a final spin at 40,000*g* for 40 min. The final supernatant was collected and used in subsequent cell viability experiments, as described above. Due to the differential growths of parental, CRISPR/Cas9 KO clones, and Arl13b:GFP clone populations, the conditioned media harvested from the faster growing cell lines were partially diluted in non-conditioned, growth factor-free media in order to normalize for the number of proliferating cells.

### Data analysis

Statistical analyses were performed in GraphPad Prism 5.0 (GraphPad Software, La Jolla, CA). Statistical tests are indicated in the text. Data in Figs. 7 and 8 were collected from at least 3 biological replicate experiments. Data in Fig. 9 were collected from technical replicates (*n* = 12 wells/group). Data in Fig. 10 were collected from technical replicates from at least 4 coverslips/group. In all analyses, *p* values that were < 0.05 were considered significant. Comparisons of groups were done using either a one-way ANOVA followed by Bonferroni post hoc test or a Student’s *t* test. All data are presented as the mean ± SEM.

## Additional files

**Additional file 1.** Confocal image of an Arl13b:GFP^+^ cell (from clone D4) immunostained for GFP (green), acetylated alpha-tubulin (blue), and PCM1 (red). Nucleus is stained with DAPI (magenta). PCM1 concentrates around the base of Arl13b:GFP^+^ cilia, which sometimes display enlarged distal tips (arrow).

**Additional file 2.** Time-lapse movie of L0 Arl13b:GFP clone D4. The nucleus in the middle undergoes mitosis and an Arl13b:GFP^+^ cilia emerges from one of the daughter cells. Nuclei are labeled with DRAQ5. Images were captured every 5 min over 23.75 h. Image exposure time = 80 ms (EGFP), 20 ms (DRAQ5).

**Additional file 3.** Time-lapse movie of L0 Arl13b:GFP clone D4. Note that the GFP^+^ cilium in the upper left center begins to turn downward, extending and retracting into the open space between the cells, and appears to make contact with neighboring cells during the process. Nuclei are labeled with DRAQ5. Images were captured every 5 min over 23.75 h. Image exposure time = 80 ms (EGFP), 20 ms (DRAQ5).

**Additional file 4.** Time-lapse movie of L0 Arl13b:GFP clone D4. Note the two cilia in the bottom right of the field of view coming in close contact. Afterwards, one of the cilia appears to detach its tip toward the end of the movie. Nuclei are labeled with DRAQ5. Images were captured every 5 min over 22.25 h. Image exposure time = 300 ms (EGFP), 20 ms (DRAQ5).

**Additional file 5.** Time-lapse movie of L0 Arl13b:GFP clone C6 (movie accompanies Fig. 3a). Note the cilium tip detaching and then breaking into smaller vesicles, as the host cell appears to round up and divide in the subsequent recording (not shown). Images were captured every 10 min over 20 h. Image exposure time = 2.5 s.

**Additional file 6.** Time-lapse movie of L0 Arl13b:GFP clone F5 (movie accompanies Fig. 3b). Note the budding of the ciliary tip which then appears to float away. Images were captured every 10 min over 24 h. Image exposure time = 1 s.

**Additional file 7.** Time-lapse movie of L0 Arl13b:GFP clone F5 (movie accompanies Fig. 3c). Note the budding of the ciliary tip which then appears to float away. Images were captured every 10 min over 24 h. Image exposure time = 1 s.

**Additional file 8.** Time-lapse movie of L0 Arl13b:GFP clone D4. Note that a cilium comes into view in the upper center and releases a vesicle from its tip that rapidly floats upward in the field of view. Nuclei are labeled with DRAQ5. Images were captured every 5 min over 22.25 h. Image exposure time = 300 ms (EGFP), 20 ms (DRAQ5).

**Additional file 9.** Time-lapse movie of L0 Arl13b:GFP clone C6. Note the excision of the ciliary tip, which then appears to float away. Images captured every 10 min over 20 h. Image exposure time = 2.5 s.

**Additional file 10.** Time-lapse movie of L0 Arl13b:GFP clone C6. Note that the cilium extends downward about 20–25 µm, appears to excise its tip, and then rapidly retracts. Images were captured every 10 min over 20 h. Image exposure time = 2.5 s.

**Additional file 11.** Time-lapse movie of L0 Arl13b:GFP clone D4. Note the excision of an approximately 1 µm-long ciliary vesicle, which then appears to float leftward. Nuclei are labeled with DRAQ5. Images were captured every 5 min over 6.75 h. Image exposure time = 300 ms (EGFP), 40 ms (DRAQ5).

**Additional file 12.** Time-lapse movie of L0 Arl13b:GFP clone D4. Towards the end of the video, the cilium in the upper left of the field of view releases a large (~ 1–2 µm in diameter) vesicle that floats away. Nuclei are labeled with DRAQ5. Images were captured every 5 min over 23.9 h. Image exposure time = 300 ms (EGFP), 20 ms (DRAQ5).

**Additional file 13.** Time-lapse movie of L0 Arl13b:GFP clone C6. Note the cilium on the left, which appears to release a ~ 5 µm-long segment of cilium that further dissociates into smaller vesicles. The remaining attached cilium then shifts off to the right, retracts, and then almost re-extends to the same length as at the beginning of the video. The cilium then appears to retract again. Images were captured every 10 min over 20 h. Image exposure time = 2.5 s.

**Additional file 14.** Characterization of cilia markers in mouse KR158 cells. The basal bodies (arrowheads) of KR158 cilia are positive for PCM1 (**A**) and gamma tubulin (gTub) (**B** and **C**), while the cilium (arrows) is positive for acetylated alpha-tubulin (aaTub), Arl13b (**B**), and type 3 adenylyl cyclase (AC3) (**C**). AC3 is also present in L0 and S3 cell cilia. Scale bars in **A**, **D** = 10 µm.

**Additional file 15.** Example of an L0 Arl13b:GFP clone D4 cell stained for aaTub. The Arl13b:GFP^+^ puncta lacks aaTub near an aaTub^+^ axoneme that is Arl13b:GFP^+^.

**Additional file 16.** CRISPR/Cas9 depletion of IFT88 and effect on ciliogenesis in L0 GBM cells. A CRISPR/Cas9 plasmid (pU6-gRNA-CMV-Cas9:2a:GFP; Sigma-Aldrich) co-expressing a GFP reporter for Cas9 and gRNA directed against human IFT88 (Target ID: HS0000334248; IFT88 gRNA target sequence: GCCATTAAATTCTACCGAA) was used to transfect parental L0 cells and generate cell clones depleted of IFT88. L0 cells were grown on 10 cm^2^ plates and transfected (Lipofectamine 2000) at 60% to 70% confluence with 0.5 μg/ml of the CRISPR/Cas9-encoding plasmid DNA. Twenty-four to 48 h after transfection, GFP^+^ cells were sorted as individual clones into 96-well plates containing 250 μl of DMEM/F12 medium supplemented with hEGF and bFGF using a BD FACS Aria II Cell Sorter. GFP^+^ clones were FAC-sorted and expanded for screening by western blot (WB) and immunostaining for acetylated alpha-tubulin^+^ cilia. (**A**) WB of L0 cell lysate showing that, compared to parental L0 (control) cells, clone C9-derived cells displayed an absence of a band for IFT88. β-Actin was used as the loading control. (**B**) Percentage of aaTub^+^ cilia in control and C9 clones. ***p < 0.001 (Student’s t test).

**Additional file 17.** Overall survival curves of low-grade glioma patients relative to low or high expression of SMO (**A**) or ARL13B (**B**). Data and statistical analyses collected from PROGgeneV2 (http://watson.compbio.iupui.edu/chirayu/proggene/database/index.php).

## Abbreviations

aaTub: acetylated alpha-tubulin
Arl13b: ADP-ribosylation factor-like protein 13b
gTub: gamma-tubulin
GBM: glioblastoma
IFT88: intraflagellar transport 88
KIF3A: kinesin family member 3A
SHH: sonic hedgehog
SMO: smoothened
