# Supplementary figures and images for: Glioma cell proliferation is enhanced in the presence of tumor-derived cilia vesicles
Source: Cilia. 2018 Nov 6;7:6. doi: 10.1186/s13630-018-0060-5 (PMC6219037; doi:10.1186/s13630-018-0060-5)

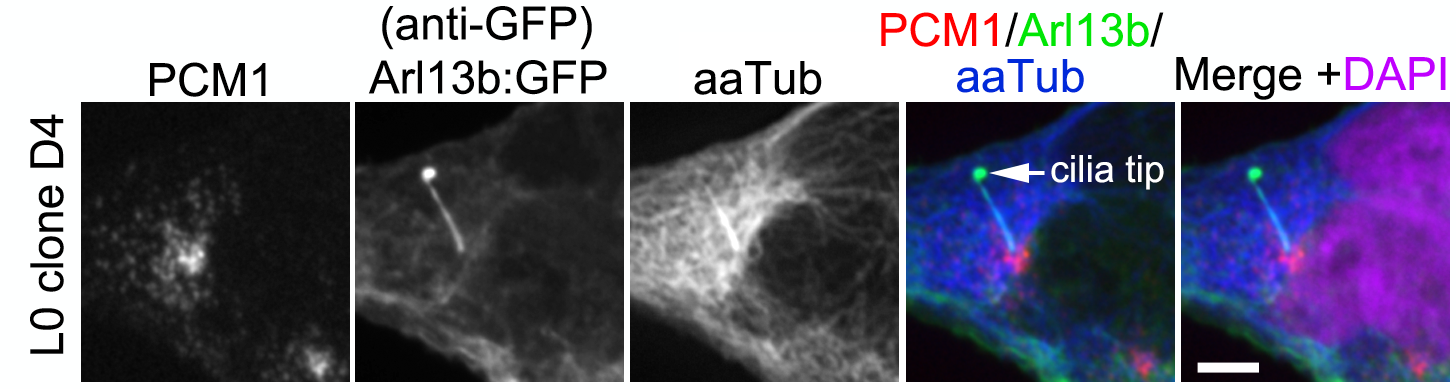

Supplement: Supplementary file 1 — Additional file 1. Confocal image of an Arl13b:GFP+ cell (from clone D4) immunostained for GFP (green), acetylated alpha-tubulin (blue), and PCM1 (red). Nucleus is stained with DAPI (magenta). PCM1 concentrates around the base of Arl13b:GFP+ cilia, which sometimes display enlarged distal tips (arrow). [file 13630_2018_60_MOESM1_ESM.tif]

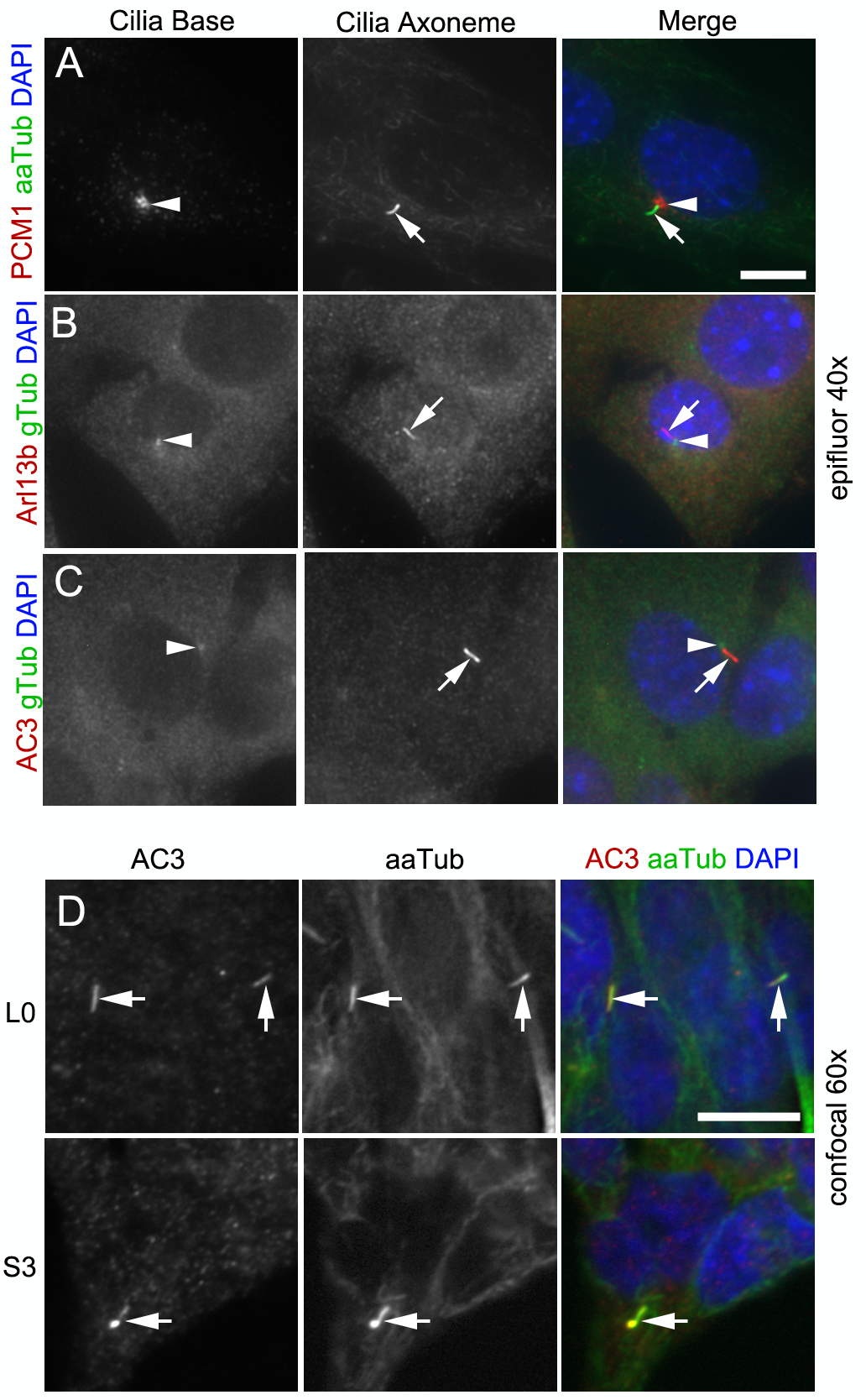

Supplement: Supplementary file 14 — Additional file 14. Characterization of cilia markers in mouse KR158 cells. The basal bodies (arrowheads) of KR158 cilia are positive for PCM1 (A) and gamma tubulin (gTub) (B and C), while the cilium (arrows) is positive for acetylated alpha-tubulin (aaTub), Arl13b (B), and type 3 adenylyl cyclase (AC3) (C). AC3 is also present in L0 and S3 cell cilia. Scale bars in A, D = 10 µm. [file 13630_2018_60_MOESM14_ESM.tif]

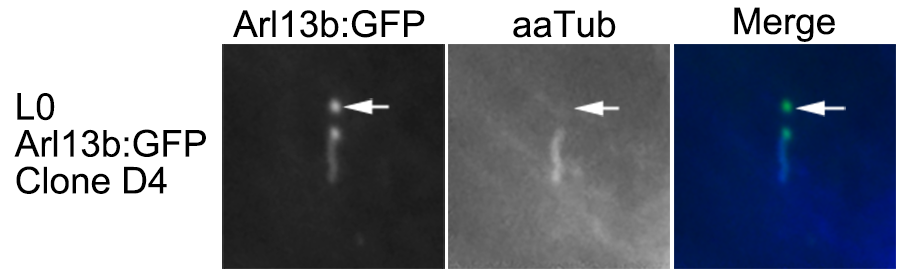

Supplement: Supplementary file 15 — Additional file 15. Example of an L0 Arl13b:GFP clone D4 cell stained for aaTub. The Arl13b:GFP+ puncta lacks aaTub near an aaTub+ axoneme that is Arl13b:GFP+. [file 13630_2018_60_MOESM15_ESM.tif]

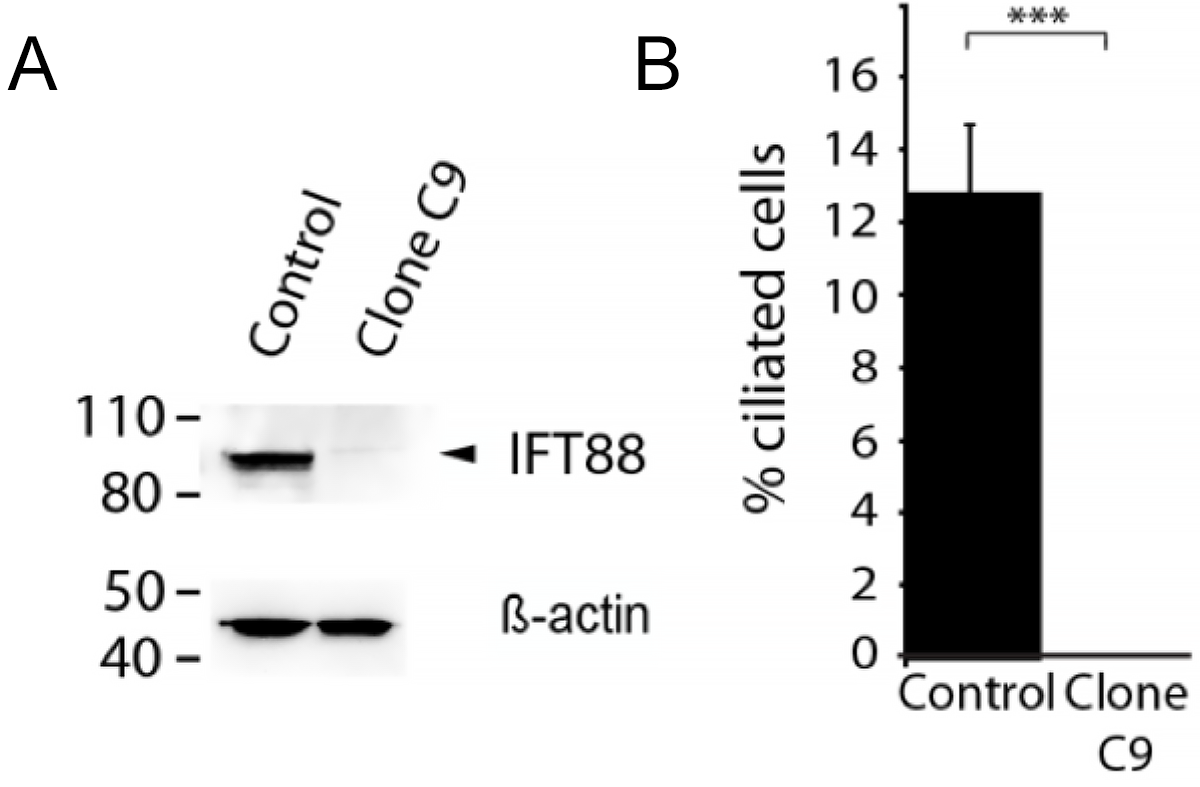

Supplement: Supplementary file 16 — Additional file 16. CRISPR/Cas9 depletion of IFT88 and effect on ciliogenesis in L0 GBM cells. A CRISPR/Cas9 plasmid (pU6-gRNA-CMV-Cas9:2a:GFP; Sigma-Aldrich) co-expressing a GFP reporter for Cas9 and gRNA directed against human IFT88 (Target ID: HS0000334248; IFT88 gRNA target sequence: GCCATTAAATTCTACCGAA) was used to transfect parental L0 cells and generate cell clones depleted of IFT88. L0 cells were grown on 10 cm2 plates and transfected (Lipofectamine 2000) at 60% to 70% confluence with 0.5 μg/ml of the CRISPR/Cas9-encoding plasmid DNA. Twenty-four to 48 h after transfection, GFP+ cells were sorted as individual clones into 96-well plates containing 250 μl of DMEM/F12 medium supplemented with hEGF and bFGF using a BD FACS Aria II Cell Sorter. GFP+ clones were FAC-sorted and expanded for screening by western blot (WB) and immunostaining for acetylated alpha-tubulin+ cilia. (A) WB of L0 cell lysate showing that, compared to parental L0 (control) cells, clone C9-derived cells displayed an absence of a band for IFT88. β-Actin was used as the loading control. (B) Percentage of aaTub+ cilia in control and C9 clones. ***p < 0.001 (Student’s t test). [file 13630_2018_60_MOESM16_ESM.tif]

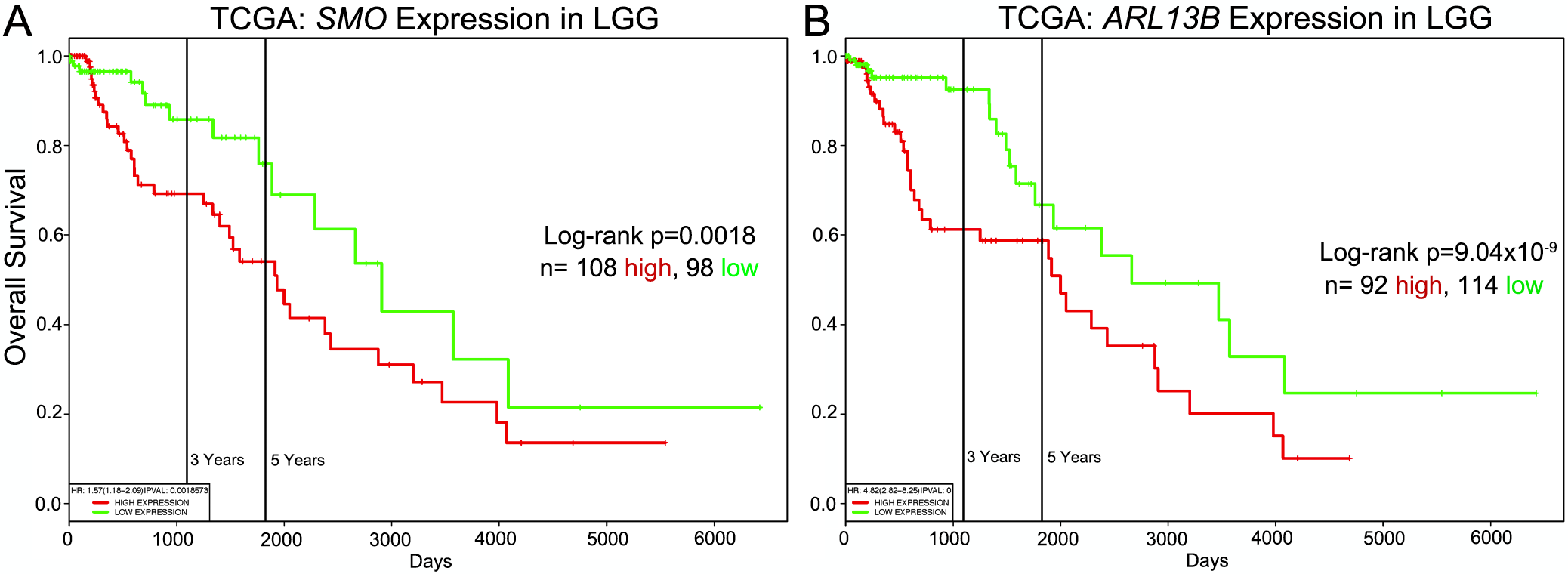

Supplement: Supplementary file 17 — Additional file 17. Overall survival curves of low-grade glioma patients relative to low or high expression of SMO (A) or ARL13B (B). Data and statistical analyses collected from PROGgeneV2 (http://watson.compbio.iupui.edu/chirayu/proggene/database/index.php). [file 13630_2018_60_MOESM17_ESM.tif]
